# *XRCC1 *and *XPD *genetic polymorphisms, smoking and breast cancer risk in a Finnish case-control study

**DOI:** 10.1186/bcr1333

**Published:** 2005-10-11

**Authors:** Katja Metsola, Vesa Kataja, Pia Sillanpää, Päivi Siivola, Liisa Heikinheimo, Matti Eskelinen, Veli-Matti Kosma, Matti Uusitupa, Ari Hirvonen

**Affiliations:** 1 Department of Industrial Hygiene and Toxicology, Finnish Institute of Occupational Health, Helsinki, Finland; 2 Department of Oncology, Kuopio University Hospital, Kuopio, Finland; 3 Department of Surgery, Kuopio University Hospital, Kuopio, Finland; 4 Department of Clinical Pathology, Kuopio University Hospital, Kuopio, Finland; 5 Department of Pathology and Forensic Medicine, University of Kuopio, Kuopio, Finland; 6 Department of Clinical Nutrition, University of Kuopio, Kuopio, Finland

## Abstract

**Introduction:**

It has been suggested that individuals with reduced DNA repair capacities might have increased susceptibility to environmentally induced cancer. In this study, we evaluated if polymorphisms in DNA repair genes *XRCC1 *(*Arg280His*, *Arg399Gln*) and *XPD *(*Lys751Gln*) modify individual breast cancer risk, with emphasis on tobacco smoking.

**Methods:**

The study population consisted of 483 incident breast cancer cases and 482 population controls of Finnish Caucasian origin. The genotypes were determined by PCR-RFLP-based methods. Odds ratio (OR) and confidence intervals (CIs) were calculated by unconditional logistic regression analyses.

**Results:**

No statistically significant overall effect in the breast cancer risk was seen for any of the studied polymorphisms. However, a significant increase in breast cancer risk was seen among ever smoking women if they carried at least one *XRCC1-399 Gln *allele (OR 2.33, 95% CI 1.30–4.19, *p*_int _0.025) or *XPD-751 Gln/Gln *genotype (OR 2.52, 95% CI 1.27–5.03, *p*_int _0.011) compared to smoking women not carrying these genotypes. The risks were found to be confined to women smoking at least five pack-years; the respective ORs were 4.14 (95% CI 1.66–10.3) and 4.41 (95% CI 1.62–12.0). Moreover, a significant trend of increasing risk with increasing number of the putative at-risk genotypes (*p *for trend 0.042) was seen. Women with at least two at-risk genotypes had an OR of 1.54 (95% CI 1.00–2.41) compared to women with no at-risk genotypes. Even higher estimates were seen for ever actively smoking women with at least two at-risk genotypes.

**Conclusion:**

Our results do not indicate a major role for *XRCC1 *and *XPD *polymorphisms in breast cancer susceptibility, but suggest that they may modify the risk especially among smoking women.

## Introduction

Breast cancer is the most common female cancer among western societies and its incidence increases constantly. Hormonal factors like early age at menarche, later age at menopause, later age at first full term pregnancy, and hormone replacement therapy are known to be the main risk factors for sporadic breast cancer [1,2]. Also, alcohol appears to contribute to the increased risk for this malignancy, whereas the results concerning smoking are inconsistent [3-6]. The inconsistencies might be due to several factors. For instance, cigarette smoke increases the production of reactive oxygen species (ROS) and contains chemical carcinogens capable of forming DNA adducts [7], both implicated in carcinogenesis. On the other hand, tobacco smoke has been suggested to have an anti-estrogenic and, therefore, anti-carcinogenic effect [8]. It has also been suggested that the genetic background might modify the association between tobacco smoke and breast cancer [4].

Unrepaired or misrepaired DNA damage can lead to gene mutations, chromosomal alterations, and genomic instability, known to have a role in cancer initiation. Accordingly, individuals with reduced DNA repair capacities might have an altered cancer risk [9-11]. During recent years, several polymorphisms in the genes encoding the DNA repair enzymes have been found and studied in relation to cancer proneness [12,13].

XRCC1 (X-ray repair cross-complementing group 1) is known to participate in base excision repair (BER) of small lesions such as oxidized or reduced bases, fragmented or nonbulky adducts, and lesions caused by methylating agents [14]. The XRCC1 is a multidomain protein that has no known catalytic activity itself but it recruits DNA polymerase β, DNA ligase III and poly(ADP-ribose) polymerase (PARP) that are needed at the site of DNA damage [15].

Three gene polymorphisms resulting in non-conservative amino acid substitutions (Arg194Trp, Arg280His and Arg399Gln) have been identified in the *XRCC1 *gene [12]. Two of these (Arg280His and Arg399Gln) were recently predicted to be likely to affect the function of the protein based on the conservation of the amino acids among protein family members [16]. The *XRCC1 Arg280His *polymorphism lies in between the DNA polymerase β and PARP binding areas [17]. The *280His *variant allele has been suggested to confer increased mutagen sensitivity [18] and breast cancer risk [19], although contrasting results exist [20-22].

The *XRCC1 Arg399Gln *polymorphism is located in the area coding for a PARP binding site. PARP is a zinc-finger containing enzyme that detects DNA strand breaks [23]. Carriers of the *XRCC1-399 Gln *variant allele have been shown to have higher levels of DNA adducts [20] and to be at greater risk for ionizing radiation sensitivity [24] and tobacco-related DNA damage [25-27]. A positive association between the *399Gln *variant allele and breast cancer risk has been seen in some studies [21,22,28,29] while no overall association has been seen in others [19,29-36].

The nucleotide excision repair (NER) pathway repairs a wide variety of DNA damage, including cross-links, oxidative damage and bulky adducts (such as polycyclic aromatic hydrocarbon (PAH)-DNA adducts). The *XPD *(XP complementation group D) gene encodes a helicase involved in transcription and in the NER pathway by unwinding of double helix at the site of deleterious DNA lesions [37]. Several single nucleotide polymorphisms (SNPs) have been described in the *XPD *gene. Two of these SNPs lead to amino acid change, Asp312Asn in exon 10 and Lys751Gln in exon 23, and are in strong linkage disequilibrium with each other [12,38]. The *XPD-751 Gln *variant allele has been associated with increased DNA adduct levels [39,40] and suboptimal DNA repair [11,38], but contrasting results also exist [41]. It has recently been associated with increased risk of smoking-related cancers, such as lung cancer [42] and squamous cell carcinoma of head and neck [43], and recently a significant effect was also seen for breast cancer [44]. Contrasting results also exist [11,40,45-47].

In this study, we evaluated the role of *XRCC1 Arg280His*, *XRCC1 Arg399Gln*, and *XPD Lys751Gln *polymorphisms in breast cancer susceptibility in our Finnish Caucasian study population. We especially aimed at examining the genotype effects in relation to smoking, which is known to cause DNA damage repaired by DNA repair enzymes of the BER and NER pathways. The role of alcohol was also evaluated in this context.

## Materials and methods

### Study population

Breast cancer cases had been referred by a physician to the Kuopio University Hospital (Finland) during the recruiting period from 1990 to 1995 because of a suspect breast sign or symptom. Women were asked to participate in the study and were interviewed by a trained study nurse before any diagnostic procedures. The study was approved by the Joint Committee of the University of Kuopio and Kuopio University Hospital. Participation was based on written consent.

A total of 516 out of 1,919 women were eventually diagnosed with histologically confirmed breast cancer. The details of the study population and recruitment have been described earlier [48]. The contact rate for the cases, calculated as described in [49], was 86%, the cooperation rate was 98%, and the overall response rate 84%.

Healthy population controls living in the same area as the cases were a randomly selected group of subjects drawn from the Finnish National Population register. They were initially contacted by a letter explaining the study protocol, and subsequently called up and invited to the hospital to be interviewed. In all, 514 controls were interviewed in parallel with the cases, all of whom agreed to participate in a genetic study. The cooperation rate for the controls was 72%.

Blood samples were collected at the time of the interview. Lymphocyte DNA was available for 483 breast cancer patients and 488 controls. Six subjects among controls were excluded, four because of earlier breast cancer diagnosis and two because of non-Finnish origin. The final case group included 483 cases (mean age 58.9 years, range 44.3 to 91.6) and 482 controls (mean age 53.5 years, range 37.5 to 77.2), all of Finnish Caucasian origin.

Detailed data on smoking habits included the amount of cigarettes with or without filter/day, years of smoking, time since quitting smoking, and time (in years) of passive smoking at home and/or at work.

### Genotyping analyses

Lymphocyte DNA (100 ng) extracted by standard methods was used as a template in PCR-based restriction fragment length polymorphism (RFLP) assays. For the *XRCC1 Arg280His *genotype determination, a 282 base pair (bp) fragment was amplified using the primers described earlier [18]. After digestion of the PCR product with 3 U of *Rsa*I, the *280Arg *wild-type allele was revealed by two 141 bp fragments, while an intact 282 bp fragment indicated the presence of the *280His *variant allele.

In the *XRCC1 Arg399Gln *genotype analysis, a 615 bp fragment amplified using the primers described in [20] was digested with 3 U of *Msp*I restriction enzyme; the *XRCC1-399 Arg *wild-type allele was revealed by the presence of 221 bp and 374 bp fragments while the *399Gln *variant allele remained intact (615 bp).

The *XPD Lys751Gln *genotypes were determined by amplifying a 324 bp fragment with the primers described in [50]. The resulting fragment was digested with 3 U of *Pst*I; the amplicon from the *751Lys *wild-type allele was cut into 220 bp and 104 bp fragments while the amplicon from the *751Gln *variant allele was cut into 157 bp, 104 bp and 63 bp fragments.

The genotype analyses were performed unaware of the case-control status. Two positive controls with known genotype and two negative controls were used within each PCR amplification batch, and two independent researchers interpreted the gel images to ensure the validity of genotyping for each polymorphism. The PCR for samples with divergent results was repeated and an additional 10% of all samples were reanalysed for each polymorphism for quality control. No discrepancies were found in the replicate tests. The *XRCC1 Arg280His *genotype could not be determined for three cases and three controls, the *Arg399Gln *genotype for four cases and four controls, and the *XPD Lys751Gln *genotype for two cases and two controls.

### Statistical analyses

Association between genotypes and risk of breast cancer were evaluated by unconditional logistic regression to calculate multivariate adjusted odds ratios (ORs) and 95% confidence intervals (CIs) using SPSS version 11.5 (SPSS Inc., Chicago, Illinois, USA). Calendar age, age at menarche, age at first full term pregnancy, number of children, history of benign breast diseases, first degree (mother, sister, daughter) family history of breast cancer, waist-to-hip ratio and use of alcohol and smoking were used as adjusting variables in all analyses. Subjects with missing values in any of the adjusting variables were excluded from the analysis. When the adjusted ORs differed significantly from the unadjusted ORs, both are shown.

Women who had smoked daily for longer than three months were considered as smokers. Never smokers were categorized as those who had never been exposed to either passive or active smoking, and as those exposed to passive smoking only. Ever smokers were further categorized according to the approximate median smoking years (15 years), daily consumption (10 cigarettes) and pack-years of smoking (5 pack-years) in the control population. Pack-years of cigarettes were calculated as the number of packs (20 cigarettes/pack) smoked per day multiplied by years of smoking.

Based on the prior knowledge of the functional significance or epidemiological evidence, the *XRCC1-280 Arg/Arg *and *XRCC1-399 Arg/Arg *genotypes were used as reference categories for all separate analyses. As the frequency of the *XRCC1-280 His *variant allele was low, the ORs were calculated for the combined *XRCC1-280 His *allele containing genotypes in all analyses. Similarly, in the stratified analyses, the *XRCC1-399 Gln *allele containing genotypes were combined to increase statistical power. In contrast, a recessive model was used for the *XPD Lys751Gln *polymorphism based on the observed genotype distributions and some earlier publications [42,51]. Therefore, the heterozygous *XPD-751 Lys/Gln *genotype was combined with the wild-type *Lys/Lys *genotype to serve as a reference category. Possible gene environment interactions were assessed using stratified analyses and interaction terms. Variables of interest were smoking and use of alcohol. Tests for interactions were assessed by the likelihood ratio test to compare goodness of fit of the model with the interaction term, to the reduced model including the main effect variable (genotype) and the main adjusting variables. All reported *p*-values are two-sided. No attempt was made to adjust for multiple comparisons.

## Results

Characteristics of the study population are shown in Table 1. Parity was associated with decreased risk of breast cancer while higher waist-to-hip ratio, first degree family history of breast cancer and history of benign breast disease were associated with increased risk. No significant associations were seen between breast cancer risk and smoking habits. Neither were any statistically significant differences seen in the mean number of cigarettes smoked per day (mean 11.2, SD 9.2, and mean 9.5, SD 6.7, for cases and controls, respectively, *p *= 0.10), or in smoking years (16.1, SD 10.8, and 14.5, SD 10.8, for cases and controls, respectively, *p *= 0.25).

The frequencies of the *XRCC1-280 His*, *XRCC1-399 Gln*, and *XPD-751 Gln *variant alleles in controls were 0.08, 0.27, and 0.43, respectively. All the genotype distributions in the control population conformed to Hardy-Weinberg equilibrium (*p *= 0.993, *p *= 0.657, and *p *= 0.876 for *XRCC1-280*, *XRCC1-399*, and *XPD-751 *locuses, respectively). No statistically significant differences were seen in the frequency of these genotypes between cases and controls (Table 2). Neither was any significant difference seen for the polymorphisms when stratified by menopausal status or age (data not shown).

When subjects were studied by the stage of the disease at the time of diagnosis, a significant increase for advanced stage (III or IV) breast cancer was seen for women with the *XRCC1-399 Gln/Gln *genotype (OR 2.86, 95% CI 1.05–7.81) compared to those with the *Arg/Arg *genotype. No increase in risk was seen for lower stage (I or II) breast cancer (OR 1.33, 95% CI 0.79–2.26). A similar tendency of increased risk for advanced stage cancer was seen for subjects with the *XRCC1-280 Arg/His *or *His/His *genotype (OR 1.99, 95% CI 0.90–4.42) compared to subjects with the *Arg/Arg *genotype. Similarly, women with the *XRCC1-399 Gln/Gln *genotype presented with a significantly increased risk for grade II and grade III tumours (OR 1.80, 95% CI 1.06–3.07) when compared to those with the *Arg/Arg *genotype, while no increase was seen for grade I tumours (OR 1.10, 95% CI 0.75–1.62). Moreover, women who carried the *XRCC1-399 Gln/Gln *genotype and were diagnosed with early stage (I or II) breast cancer had tumours of higher grade (II or III) marginally (*p *= 0.065, one-sided) more often than those with the *Arg/Arg *genotype, 86.1% (31/36) versus 72.5 % (137/189), respectively.

When the association between *XRCC1-399 *genotypes and breast cancer risk was studied according to smoking habits, increased breast cancer risk with dose-response was seen among women who had ever smoked actively and carried either one (OR 2.14, 95% CI 1.15–3.97) or two (OR 3.27, 95% CI 1.25–8.58, *p *for trend = 0.003) *XRCC1-399 Gln *variant alleles compared to those carrying the *Arg/Arg *genotype (*p *for interaction between smoking habits and *XRCC1-399 *genotype 0.025) (Table 3). A similar increase in risk was seen for ever smoking women with the *XPD-751 Gln/Gln *genotype compared to ever smoking women without this genotype (OR 2.52, 95% CI 1.27–5.03, *p *for interaction 0.011).

When ever smoking women were further stratified by pack-years smoked (<5, ≥5 pack-years), the increase in risk was seen to be confined to those who had smoked over five pack-years and carried at least one *XRCC1-399 Gln *allele (OR 4.14, 95% CI 1.66–10.3), or the *XPD-751 Gln/Gln *genotype (OR 4.41, 95 % CI 1.62–12.0) compared to similarly smoking women without these genotypes (Table 4). Similar effects were seen for the *XRCC1 Arg399Gln *genotypes when smokers were stratified by daily tobacco consumption (<10, ≥10 cigarettes/day) or by smoking years (<15, ≥15 years). The ORs were 5.32 (95% CI 1.97–14.4) for women smoking ≥10 cigarettes/day and carrying at least one *XRCC1-399 Gln *allele, and 4.03 (95% CI 1.40–11.6) for women who had smoked ≥15 years and carried at least one *XRCC1-399 Gln *allele, compared to women with similar smoking habits but with the *XRCC1-399 Arg/Arg *genotype. For the carriers of the *XPD-751 Gln/Gln *genotype, a similar increase in the risk of breast cancer was seen for women smoking ≥10 cigarettes/day (OR 4.78, 95% CI 1.50–15.2) while no statistically significant increase was seen by smoking years (OR 2.25, 95% CI 0.85–5.96 for women smoking ≥15 years), compared to women smoking the same amount, but not carrying the homozygous variant *XPD-751 Gln/Gln *genotype.

When stratified by current use of alcohol, women who reported using alcohol weekly to daily and carried the *XPD-751 Gln/Gln *genotype were at 3.18-fold (95% CI 1.34–7.57) increased risk of breast cancer compared to similarly drinking women carrying the other genotypes (*p *for interaction 0.026). No interaction was found between *XRCC1-280 *or *XRCC1-399 *genotypes and current use of alcohol (data not shown).

When the joint effect of the *XRCC1-280*, *XRCC1-399*, and *XPD-751 *genotypes was studied, a statistically significant increase in the risk of breast cancer was seen for subjects carrying two at-risk genotypes of these genes (OR 1.54, 95% CI 1.00–2.37) compared to subjects with the wild-type genotypes for all three polymorphic sites (Table 5). This increase was mainly due to the combined effect of *XRCC1-399 *and *XPD-751 *genotypes (OR 1.80, 95% CI 1.05–3.08, *p *for gene-gene interaction 0.043). A trend of increasing risk with increasing number of at-risk genotypes was seen (*p *for trend 0.042). However, this estimate did not reach statistical significance (OR 4.76, 95% CI 0.48–47.8), possibly due to the low number of subjects with all the three at-risk genotypes (four cases and one control). When the combined effects were studied among ever active smokers, women who carried any two at-risk genotypes were at remarkably increased risk of breast cancer; the adjusted OR was 10.7 (95% CI 3.62–31.6) compared to those without these genotypes (Table 5). This effect was mainly confined to combination of *XRCC1-399 *and *XPD-751 *at-risk genotypes (OR 12.1, 95% CI 3.52–41.5). When the combined effect was calculated for the number of at risk alleles (*XRCC1-280 His*, *XRCC1-399 Gln *and *XPD-751 Gln*), a similar increase in the risk was seen; subjects with three at-risk alleles had an OR of 1.72 (95% CI 1.03–2.87; *p *for trend 0.069) among all women, and OR 4.62 (95% CI 1.56–13.7; *p *for trend 0.01) among ever actively smoking women compared to women with no at-risk alleles. Only four cases and one control carried simultaneously four at-risk alleles, and none more than four (of the six).

## Discussion

In this study, we examined the role of *XRCC1 Arg280His*, *XRCC1 Arg399Gln *and *XPD Lys751Gln *polymorphisms in relation to breast cancer risk in a Finnish study population. As the products of these genes act in BER and NER pathways, and as some evidence exists on the association of these polymorphisms with smoking-related cancers [13,42,43,52], our special interest was to study the role of these DNA repair enzymes among smoking women. The hypothesis was also supported by a recent finding of an association between the *XPD-751 Gln/Gln *genotype and breast cancer risk in smoking women [44].

The two polymorphisms *Arg280His *and *Arg399Gln *in the coding region of the *XRCC1 *gene were recently predicted to be 'possibly damaging' to *XRCC1 *function based on the conservation of the sequences in mammalian orthologues [16]. In agreement with this, the frequency of the variant *XRCC1-399 Gln *allele was somewhat higher among the present cases compared to controls, leading to a tendency of increased breast cancer risk. A similar effect has been reported in studies among Korean [28], US radiologic technologists [21], Indian [22], and African-American [29] women. No increased risk was found for white American women [29], in agreement with three other studies performed among American women [30-33]. Moreover, no association was seen in studies among Chinese [35], French [19], Canadian [34], Turkish [36] and Danish [46] women.

In contrast to the *XRCC1 Arg399Gln *polymorphisms, the *Arg280His *polymorphism did not significantly modify breast cancer risk in the present study. Similarly, no association was seen for Indian women [22] or for US radiologic technologists [21]. On the other hand, our findings are in contrast to a French study showing a 1.8-fold (95% CI 1.04–3.08) increase in breast cancer risk for the *XRCC1-280 Arg/His *genotype [19]. One reason for this divergence could be the lack of power; the frequency of the *280His *allele is low (0.08) among Caucasians, including Finns. Consequently, even though having almost twice the size of the French study, the power of our study to detect an OR of 1.5 at a 0.05 significance level was only 67% for the *XRCC1-280 His *allele containing genotypes. As the *XRCC1-194 Trp *variant allele is even less frequent among Finns (approximately 0.03) [45] compared to other Caucasians, and as the amino acid change has not been shown to affect protein function [16], we decided not to include this polymorphism in our study.

The *XPD Lys751Gln *polymorphism has been suggested to be the most important functional polymorphism in the gene due to major change in the electronic configuration of the respective amino acid in an important interaction domain of the protein [53]. However, no significant overall association with breast cancer was seen in our study for the *XPD Lys751Gln *genotypes. This was in agreement with the other five studies on the *XPD Lys751Gln *polymorphism and breast cancer risk including one in Finnish [45], one in Danish [46], one in German [47], and two in US Caucasian women [11,40]. In contrast, a significant association between the *XPD-751 Gln *allele and breast cancer risk was seen in a recent study among American women [44]. Moreover, the *XPD Asp312Asn *polymorphism was recently shown to be associated with breast cancer risk in a German population [47]. We decided not to analyse the *XPD Asp312Asn *polymorphism as it has been shown to be strictly linked with the *Lys751Gln *polymorphism [12,38].

When the present study subjects were stratified by stage of disease, the *XRCC1-399 Gln *allele posed an elevated risk for more advanced stage breast cancer. A similar tendency of increased risk for more advanced stage breast cancer was also seen for the *Arg280His *polymorphism. It can be hypothesized that defective DNA repair leads to more aggressive and, therefore, more advanced tumours at the time of diagnosis. This was also supported by the association of the *XRCC1-399 Gln *allele with higher grade tumours. However, as earlier studies on breast cancer have not evaluated the genotype effects by the stage of the disease or tumour grade, these findings remain to be confirmed in future studies.

Smoking alone did not significantly affect breast cancer risk in the present study. This is in agreement with the majority of epidemiological studies on smoking and breast cancer risk, as well as with a recent report of the Collaborative Group on Hormonal Factors in Breast cancer, which concluded that cigarette smoking has little or no effect on the risk of developing breast cancer [3]. There are, however, some studies reporting increased risk in special subgroups, such as women who started to smoke at an early age or before first pregnancy, women smoking high intensity or long duration, passively smoking women, and women with specific genotypes (reviewed in [4]). In our study, a significant interaction was seen between smoking habits and the *XRCC1 Arg399Gln *(*p *= 0.025) or *XPD Lys751Gln *(*p *= 0.011) genotypes. Subjects with the variant *Gln/Gln *genotypes were at increased risk of developing breast cancer if they had ever smoked. Furthermore, a gene-dosage effect was seen for the *XRCC1 Arg399Gln *genotype; the increased risk was higher for subjects carrying two *XRCC1-399 Gln *alleles. In contrast, the effect of the *XPD-751 Gln *allele seemed to be recessive. When ever smokers were further stratified by the amount of smoking, the risk estimates remained statistically significant among those who reported having smoked more than five pack-years.

The associations found between the *XRCC1 Arg399Gln *and/or *XPD Lys751Gln *genotypes, smoking, and breast cancer risk are biologically plausible. Cigarette smoke is a rich source of chemical carcinogens and ROS [7]. ROS can initiate lipid peroxidation, oxidize proteins, and cause base damage and DNA single strand breaks that are repaired through BER. Accordingly, the *XRCC1-399 Gln/Gln *genotype has been found to be related to increased sister chromatid exchange frequencies among smokers [25]. Similarly, a higher frequency of sister chromatid exchange has been reported for carriers of the *399Gln *allele who have smoked ≥10 cigarettes/day [27], and higher levels of tobacco-specific nicotine-derived nitrosamino ketone-induced sister chromatid exchange in cells with the *XRCC1-*399 *Gln *allele containing genotypes compared to cells homozygous for the *Arg *allele [26]. It can thus be hypothesized that the *XRCC1-399 Gln *allele is associated with increased risk of smoking-dependent cancers. In agreement with this, a higher risk of lung cancer has been reported for the carriers of the *XRCC1 Gln/Gln *genotype [52], although negative studies also exist [54,55]. Breast cancer is not generally regarded as a smoking-dependent cancer. In a recent study on breast cancer, the *XRCC1-399 Gln *allele was significantly associated with detectable PAH-adducts only in never smokers [33]. They speculated that smoking might stimulate DNA repair and thus the effect seen would be due to other sources of PAHs. Only one earlier study on breast cancer risk has reported a positive association between *XRCC1-399 *polymorphism and smoking, but in contrast to our study, the highest risk was seen for subjects who were homozygous for the *XRCC1-399 Arg *allele [29]. Unfortunately, the possible association with smoking has not been reported in all studies on the *XRCC1-399 *genotypes [19,22,30,31,36].

In addition to ROS, tobacco smoke includes chemical carcinogens, such as polycyclic aromatic hydrocarbons, aromatic amines and tobacco-specific nitrosamines, that can produce bulky DNA adducts, which are repaired through the NER pathway. There is evidence that tobacco smoke constituents can reach breast tissue [7], and higher levels of the tobacco smoke derived 4-aminobiphenyl adducts [56] and PAH-DNA adducts have been found in breast tissue of breast cancer cases [57-59]. Mammary epithelial cells are capable of metabolising and activating these compounds. Therefore, the hypothesis is that breast cancer cases with suboptimal DNA repair capacity would have less efficient removal of these adducts, thus making them more vulnerable to the hazardous effects of these compounds. Accordingly, the *XPD-751 Gln *allele has been associated with increased DNA adduct levels in never smokers [39] and in tumour tissue from breast cancer cases [40], as well as with suboptimal DNA repair [11,38]. A recent meta-analysis showed an association between the *XPD-751 Gln/Gln *genotype and increased lung cancer risk [42]. This was not, however, confirmed by another recent meta-analysis [60]. In accordance with our results, a similar increased breast cancer risk was recently seen among currently smoking American women with the *XPD-751 Gln/Gln *genotype [44]. Similarly to our study, no clear pattern with duration of smoking was seen. This could partly be due to low numbers in these strata, or it can be hypothesized that a higher intensity of smoking is needed to see the difference between these genotypes, while in subjects with lower intensity but longer duration the repair system is capable of overcoming the harmful effects.

Alcohol is a known risk factor for breast cancer; increased estrogen and androgen levels have been observed in alcohol consuming women [61]. Alcohol consumption is also believed to contribute to oxidative stress [62]. We therefore analysed the results also stratified by the use of alcohol. A significantly increased three-fold risk of breast cancer was seen for women who carried the *XPD-751 Gln/Gln *genotype and used alcohol weekly or daily. No earlier studies on the *XPD *genotype and breast cancer have reported an association with the use of alcohol. However, a 2.59-fold (95% CI 1.25–5.34) risk was seen for squamous cell carcinoma of head and neck among current drinkers carrying the *XPD-751 Gln/Gln *genotype compared to those drinking the same amount but with the other genotypes [43].

When the gene-gene interactions were studied, clear combined effects were seen, especially among ever smoking women; smoking women with any two at-risk genotypes were at remarkably increased risk of breast cancer (OR 10.7, 95% CI 3.62–31.6) compared to smoking women without these genotypes. These effects were mainly due to *XRCC1-399 *and *XPD-751 *genotypes while the *XRCC1-280 *genotype was a minor contributor, possibly partly due to the very low frequency of the *XRCC1-280His *variant alleles. When the combined effect was studied according to the number of at-risk alleles, subjects with any three variant alleles were found to be at increased risk. There was no subject who simultaneously carried more than four at-risk alleles in these three polymorphic sites.

It should be noted that even if our study involved a reasonable number of study subjects, the numbers in some subgroup analyses were small, and the statistical power to detect significant point-estimates is, therefore, low. Furthermore, due to multiple comparisons performed in the study, the possibility of chance findings should be borne in mind. However, in addition to the plausibility of the findings, the dose-response seen by the number of at-risk alleles or genotypes and amounts smoked support the causality of the findings.

In case-control studies, the possibility of selection and recall bias should be considered. However, the detailed data about smoking habits and other life style factors were collected prior to diagnosis, and the exposure to passive smoking, quite often disregarded, was taken into account. It is also unlikely that selection would be related to an individual's genotype. As the statistical analyses were performed within exposure groups and not between them, the possibility of biased estimates is further reduced.

Even though the present findings are biologically plausible, the possibility of the association with another gene polymorphism in the same region should be considered. Accordingly, it was recently reported that three markers, *RAI*, *ASE-1 *and *ERCC1*, in the same chromosomal region 19q13.2-3 define a high-risk haplotype that poses women under 55 years to a significantly increased breast cancer risk [46]. In that study, no association was seen with the *XRCC1 *or *XPD *genotypes but the effects were not studied by smoking habits. In our study, no difference in the risk estimates was seen by age.

Finally, it should be noted that not all the known polymorphisms in the BER and NER pathways were analysed in this study. For the *XRCC1 Arg194Trp *polymorphism, association has been seen with increased mutagen sensitivity [63], risk of developing an adverse response to radiotherapy [19], as well as interaction with high intake of fruits and vegetables [33], and it could thus be hypothesized to affect breast cancer risk in population where it exists in higher frequency. Similarly, polymorphism has been detected in another gene, *OGG1*, encoding an enzyme important in the BER pathway, as well as numerous other genes encoding for possibly important repair enzymes in the NER pathway, namely *XPA*, *XPC*, *XPF *and *XPG*.

## Conclusion

We found a statistically significant association between the *XRCC1 *and *XPD *genotypes and breast cancer risk in Finnish smoking women, especially when enzymes in both DNA repair pathways were defective. This finding is of particular interest as smoking, being the main preventable risk factor for cancer, is constantly increasing among female adolescents in many countries, including Finland. However, it should be borne in mind that the two studied DNA repair genes account for only a small part of the known genetic variations in these pathways and, therefore, most probably do not explain the whole picture, but should be considered in the context of other possible genes. These results, together with our earlier results concerning polymorphisms in estrogen metabolizing enzymes [64], support the view that it is crucial to study simultaneously the effect of environmental exposures and genotypes in order to better understand and reveal the complex associations between genetic factors, life style and cancer.

## Abbreviations

BER = base excision repair; bp = base pair; CI = confidence interval; NER, nucleotide excision repair; OR = odds ratio; PARP = poly(ADP-ribose)polymerase; PCR = polymerase chain reaction; RFLP = restriction fragment length polymorphism; ROS = reactive oxygen species.

## Competing interests

The authors declare that they have no competing interests.

## Authors' contributions

KM was responsible for supervising the laboratory work, for performing statistical analyses, and for manuscript preparation. VK participated in the design and coordination of the study, helped to draft the manuscript and acts as the principal clinician in the study group. PS, PS and LH carried out the genotyping analyses and drafted the manuscript. ME participated in the design and coordination of the study and was clinical coordinator of collection of study patient material. VMK performed histopathological analyses of all cases, and helped to draft the manuscript. MU has been involved in the planning of the study, collection of clinical data, in particular with respect to the genetic part of the study, and he has commented on the manuscript in the writing phase. AH participated in the design and coordination of the molecular epidemiology part of the study, was responsible for acquisition of funding for the molecular epidemiology part, and was involved in the preparation of the manuscript. All authors read and approved the final manuscript.

## Figures and Tables

**Table 1:** Selected characteristics of the study subjects

Characteristic	Case/control^a^	OR^b ^(95% CI)
Age at menarche		
≤12	98/101	1.0
13–14	219/251	0.82 (0.59–1.16)
≥15	150/127	0.99 (0.68–1.46)
Age at first full-term pregnancy		
Nulliparous	102/57	1.0
≤25	237/263	0.55 (0.38–0.81)
26–30	94/122	0.44 (0.29–0.69)
≥31	47/40	0.64 (0.36–1.12)
Number of full-term pregnancies		
Nulliparous	102/57	1.0
1	68/64	0.59 (0.36–0.98)
2	141/181	0.50 (0.33–0.76)
3+	171/180	0.54 (0.36–0.80)
Waist-to-hip ratio		
≤0.91	187/236	1.0
>0.91	291/243	1.38 (1.06–1.81)
First-degree family history of breast cancer		
No	424/459	1.0
Yes	54/22	2.53 (1.48–4.31)
History of benign breast disease		
No	296/313	1.0
Yes	180/167	1.33 (1.01–1.75)
Current alcohol intake		
Never	271/206	1.0
Once a month or less	134/187	0.74 (0.54–1.01)
Daily-weekly	75/89	0.87 (0.59–1.29)
Smoking habits		
Never active or passive	210/182	1.0
Passive only	153/169	0.78 (0.58–1.06)
Ever active	112/130	0.91 (0.65–1.28)
<5 pack-years	45/63	0.75 (0.48–1.16)
≥5 pack-years	67/67	1.08 (0.72–1.62)

^a ^Total number of cases and controls does not correspond because of missing values.

^b ^Adjusted for age. CI, confidence interval; OR, odds ratio.

**Table 2:** Association between *XRCC1 *and *XPD *polymorphisms and breast cancer risk

Genotype	Cases (%)	Controls (%)	OR (95% CI)^a^
*XRCC1-280*			
*Arg/Arg*	399 (83.1)	406 (84.8)	1.0
*Arg/His*	78 (16.3)	70 (14.6)	
*His/His*	3 (0.6)	3 (0.6)	1.15 (0.80–1.66)^b^
*XRCC1-399*			
*Arg/Arg*	237 (49.5)	256 (53.6)	1.0
*Arg/Gln*	196 (40.9)	185 (38.7)	1.24 (0.93–1.65)
*Gln/Gln*	46 (9.6)	37 (7.7)	1.39 (0.84–2.29)^c^
*Arg/Gln + Gln/Gln*	242 (50.5)	222 (46.4)	1.26 (0.96–1.66)
*XPD-751*			
*Lys/Lys*	147 (30.6)	155 (32.3)	1.0
*Lys/Gln*	238 (49.5)	237 (49.4)	1.03 (0.76–1.40)
*Gln/Gln*	96 (20.0)	88 (18.3)	1.10 (0.74–1.63)
*Lys/Lys+Lys/Gln*	385 (80.0)	325 (81.7)	1.0
*Gln/Gln*	96 (20.0)	88 (18.3)	1.08 (0.77–1.53)

^a^Odds ratios (ORs) and confidence intervals (CIs) adjusted for age, age at menarche, age at first full term pregnancy, number of pregnancies, history of benign breast disease, first degree family history of breast cancer, weist-to-hip ratio, smoking and use of alcohol. ^b^OR for *Arg/His *and *His/His *genotypes combined. ^c ^*p *for trend = 0.105.

**Table 3:** Association between *XRCC1 *and *XPD *genotypes and breast cancer risk according to smoking habits

Genotype	Never active or passive smoking			Only passive smoking			Active smoking		
	Cases (%)	Controls (%)	OR (95% CI)^a^	Cases (%)	Controls (%)	OR (95% CI)^a^	Cases (%)	Controls (%)	OR (95% CI)^a^

*XRCC1-280*									
*Arg/Arg*	177 (84.7)	154 (84.6)	1.0	120 (79.5)	140 (83.8)	1.0	94 (83.9)	111 (86.0)	1.0
*Arg/His+His/His*	32 (15.3)	28 (15.4)	1.09 (0.60–1.99)	31 (20.5)	27 (16.2)	1.11 (0.59–2.08)	18 (16.1)	18 (14.0)	1.41 (0.65–3.08)
*XRCC1-399*									
*Arg/Arg*	118 (56.5)	89 (48.9)	1.0	72 (47.7)	91 (54.5)	1.0	45 (40.5)	75 (58.6)	1.0
*Arg/Gln*	76 (36.4)	76 (41.8)	0.83 (0.53–1.31)	67 (44.4)	66 (39.5)	1.40 (0.84–2.32)	49 (44.2)	43 (33.6)	2.14 (1.15–3.97)
*Gln/Gln*	15 (7.2)	17 (9.3)	0.73 (0.33–1.64)	12 (7.9)	10 (6.0)	1.61 (0.61–4.23)	17 (15.3)	10 (7.8)	3.27 (1.25–8.58)^b^
*Arg/Gln+Gln/Gln*	91 (43.5)	93 (51.1)	0.81 (0.53–1.25)	79 (52.3)	76 (45.5)	1.42 (0.87–2.32)	66 (59.5)	53 (41.4)	2.33 (1.30–4.19)^c^
*XPD-751*									
*Lys/Lys*	66 (31.6)	58 (31.9)	1.0	40 (26.3)	56 (33.3)	1.0	40 (35.7)	40 (31.0)	1.0
*Lys/Gln*	109 (52.2)	91 (50.0)	1.03 (0.63–1.66)	83 (54.6)	77 (45.8)	1.35 (0.78–2.34)	40 (35.7)	77 (45.8)	0.68 (0.35–1.33)
*Gln/Gln*	34 (16.3)	33 (18.1)	0.78 (0.40–1.49)	29 (19.1)	35 (20.8)	0.84 (0.41–1.69)	32 (28.6)	35 (20.8)	1.96 (0.89–4.32)
*Lys/Lys+Lys/Gln*	175 (83.7)	149 (81.9)	1.0	123 (80.9)	133 (79.2)	1.0	80 (71.4)	109 (84.5)	1.0
*Gln/Gln*	34 (16.3)	33 (18.1)	0.77 (0.43–1.39)	29 (19.1)	35 (20.8)	0.70 (0.38–1.29)	32 (28.6)	20 (15.5)	2.52 (1.27–5.03)^d^

^a^Odds ratios (ORs) and confidence intervals (CIs) adjusted for age, age at menarche, age at first full term pregnancy, number of pregnancies, history of benign breast disease, first degree family history of breast cancer, weist-to-hip ratio and use of alcohol. ^b^*p *for trend = 0.003. ^c^Interaction between smoking habits and *XRCC1-399 *genotype (*p *= 0.025). ^d^Interaction between smoking habits and *XPD-751 *genotype (*p *= 0.011).

**Table 4:** The association between *XRCC1 *and *XPD *genotypes and breast cancer risk according to pack-years smoked

	<5 pack-years			>5 pack-years		
	Cases n (%)	Controls n (%)	OR (95% CI)^a^	Cases n (%)	Controls n (%)	OR (95% CI)^a^

*XRCC1-280*						
*Arg/Arg*	37 (82.2)	51 (82.3)	1.0	57 (85.1)	60 (89.6)	1.0
*Arg/His+His/His*	8 (17.8)	11 (17.7)	0.91 (0.28–2.95)	10 (14.9)	7 (10.4)	1.99 (0.57–7.02)
*XRCC1-399*						
*Arg/Arg*	20 (44.4)	35 (57.4)	1.0	25 (37.9)	40 (59.7)	1.0
*Arg/Gln*	18 (40.0)	22 (36.1)	1.21 (0.46–3.18)	31 (47.0)	21 (31.3)	4.31 (1.66–11.2)
*Gln/Gln*	7 (15.6)	4 (6.6)	3.99 (0.82–19.4)	10 (15.2)	6 (9.0)	3.55 (0.81–15.6)
*Arg/Gln+Gln/Gln*	25 (55.6)	26 (42.6)	1.61 (0.64–4.06)	41 (62.1)	27 (40.3)	4.14 (1.66–10.3)
*XPD-751*						
*Lys/Lys*	18 (40.0)	20 (32.3)	1.0	22 (32.8)	20 (29.9)	1.0
*Lys/Gln*	18 (40.0)	31 (50.0)	1.01 (0.37–2.77)	22 (32.8)	38 (56.7)	0.40 (0.14–1.12)
*Gln/Gln*	9 (20.0)	11 (17.7)	1.59 (0.44–5.74)	23 (34.3)	9 (13.4)	2.77 (0.90–8.59)
*Lys/Lys+Lys/Gln*	36 (80.0)	51 (82.3)	1.0	44 (65.7)	58 (86.6)	1.0
*Gln/Gln*	9 (20.0)	11 (17.7)	1.45 (0.46–4.56)	23 (34.3)	9 (13.4)	4.41 (1.62–12.0)

^a^Odds ratios (ORs) and confidence intervals (CIs) adjusted for age, age at menarche, age at first full term pregnancy, number of pregnancies, history of benign breast disease, first degree family history of breast cancer, weist-to-hip ratio and use of alcohol.

**Table 5:** Combined *XRCC1 *and *XPD *genotypes and breast cancer risk among all and ever smoking women

				All women			Ever actively smoking women		
No of at risk genotypes	*XRCC1-280*	*XRCC1-399*	*XPD-751*	Case/Control	Unadjusted OR (95% CI)	Adjusted OR (95% CI)^a^	Case/Control	Unadjusted OR (95% CI)	Adjusted OR (95% CI)^a^

0	*Arg/Arg*	*Arg/Arg*	*Lys/Lys+ Lys/Gln*	145/159	1.0	1.0	23/46	1.0	1.0
1	*Arg/Arg*	*Arg/Arg*	*Gln/Gln*	32/44	0.80 (0.48–1.33)	0.72 (0.42–1.25)	8/12	1.33 (0.48–3.72)	1.20 (0.37–3.86)
1	*Arg/Arg*	*Arg/Gln+ Gln/Gln*	*Lys/Lys+ Lys/Gln*	172/170	1.11 (0.81–1.51)	1.15 (0.83–1.60)	43/48	1.79 (0.94–3.43)	1.93 (0.93–4.05)
1	*Arg/His+His/His*	*Arg/Arg*	*Lys/Lys+ Lys/Gln*	45/44	1.12 (0.70–1.80)	1.19 (0.72–1.97)	11/15	1.47 (0.58–3.70)	1.94 (0.69–5.44)
1 (any one at risk genotype)	*-*	*-*	*-*	249/258	1.06 (0.80–1.41)	1.09 (0.81–1.47)	62/75	1.65 (0.91–3.02)	1.80 (0.91–3.56)
2	*Arg/Arg*	*Arg/Gln+ Gln/Gln*	*Gln/Gln*	46/32	1.58 (0.95–2.61)	1.80 (1.05–3.08)	19/5	7.60 (2.52–22.0)	12.1 (3.52–41.5)
2	*Arg/His+ His/His*	*Arg/Gln+ Gln/Gln*	*Lys/Lys+ Lys/Gln*	19/18	1.16 (0.59–2.29)	1.28 (0.62–2.64)	3/0	-	-
2	*Arg/His+ His/His*	*Arg/Arg*	*Gln/Gln*	13/9	1.58 (0.66–3.82)	1.36 (0.52–3.59)	3/2	3.00 (0.47–19.2)	3.47 (0.44–27.1)
2 (any two at risk genotypes)	*-*	*-*	*-*	78/59	1.45 (0.97–2.18)	1.54 (1.00–2.37)	25/7	7.14 (2.69–19.0)	10.7 (3.62–31.6)
3	*Arg/His+His/His*	*Arg/Gln+ Gln/Gln*	*Gln/Gln*	4/1	4.39 (0.49–39.7)	4.76 (0.48–47.8)^b^	1/0	-	-

^a^Odds ratios (ORs) and confidence intervals (CIs) adjusted for age, first degree family history of breast cancer, age at menarche, number of children, age at first full term pregnancy, history of benign breast disease, weist-to-hip ratio and use of alcohol. ^b^*p *for trend of having 1, 2 or 3 at-risk genotypes 0.042.
